# Global Analysis of the Zinc Homeostasis Network in *Pseudomonas aeruginosa* and Its Gene Expression Dynamics

**DOI:** 10.3389/fmicb.2021.739988

**Published:** 2021-10-08

**Authors:** Verena Ducret, Melina Abdou, Catarina Goncalves Milho, Sara Leoni, Oriane Martin--Pelaud, Antoine Sandoz, Inés Segovia Campos, Mary-Lou Tercier-Waeber, Martina Valentini, Karl Perron

**Affiliations:** ^1^Microbiology Unit, Department of Botany and Plant Biology, University of Geneva, Geneva, Switzerland; ^2^Department of Inorganic and Analytical Chemistry, University of Geneva, Geneva, Switzerland; ^3^Department of Earth Sciences, University of Geneva, Geneva, Switzerland; ^4^Department of Microbiology and Molecular Medicine, CMU, Faculty of Medicine, University of Geneva, Geneva, Switzerland; ^5^Institute of Pharmaceutical Sciences of Western Switzerland, University of Geneva, Geneva, Switzerland

**Keywords:** zinc, homeostasis, NanoString, carbapenem, *Pseudomonas aeruginosa*

## Abstract

Zinc is one of the most important trace elements for life and its deficiency, like its excess, can be fatal. In the bacterial opportunistic pathogen *Pseudomonas aeruginosa*, Zn homeostasis is not only required for survival, but also for virulence and antibiotic resistance. Thus, the bacterium possesses multiple Zn import/export/storage systems. In this work, we determine the expression dynamics of the entire *P. aeruginosa* Zn homeostasis network at both transcript and protein levels. Precisely, we followed the switch from a Zn-deficient environment, mimicking the initial immune strategy to counteract bacterial infections, to a Zn-rich environment, representing the phagocyte metal boost used to eliminate an engulfed pathogen. Thanks to the use of the NanoString technology, we timed the global silencing of Zn import systems and the orchestrated induction of Zn export systems. We show that the induction of Zn export systems is hierarchically organized as a function of their impact on Zn homeostasis. Moreover, we identify PA2807 as a novel Zn resistance component in *P. aeruginosa* and highlight new regulatory links among Zn-homeostasis systems. Altogether, this work unveils a sophisticated and adaptive homeostasis network, which complexity is key in determining a pathogen spread in the environment and during host-colonization.

## Introduction

Zinc (Zn), commonly found as the divalent cation Zn^2+^, is a trace element primarily involved as a cofactor of many enzymes and therefore essential for life. It is considered as the second most important trace metal after iron (Fe) (Blencowe and Morby, 2003). In prokaryotes, Zn is principally found bound to proteins (approximately 6% of the proteome) and the free cellular fraction is kept at a concentration as low as femtomolar (Outten and O’Halloran, 2001). In excess, this metal becomes toxic, especially by competing with other trace elements, giving rise to protein mismetallization (Foster et al., 2014). The equilibrium of cellular Zn concentration is therefore tightly regulated and numerous systems involved in Zn homeostasis (and that of other divalent cations) are well-described in several bacteria (Chandrangsu et al., 2017).

**FIGURE 1:**
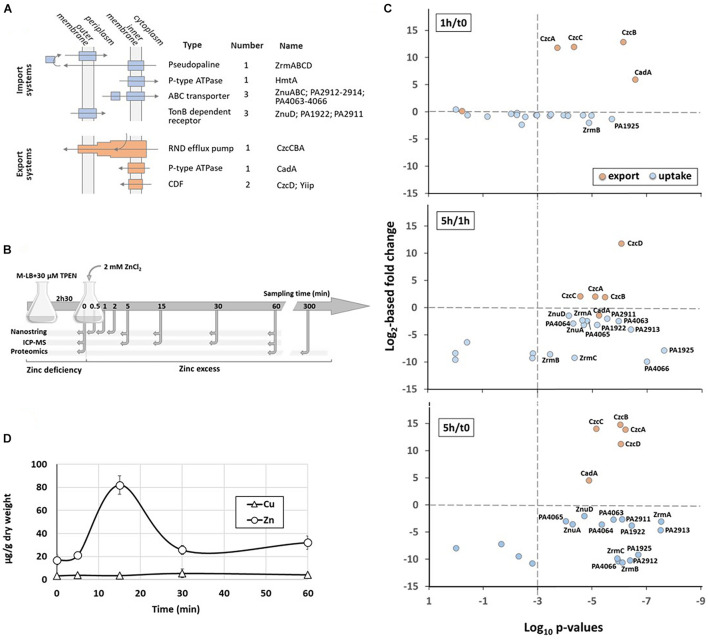
Dynamics of Zn homeostasis in *P. aeruginosa.* **(A)** The four families of Zn import systems (in blue) and the 3 families of Zn export systems (in orange) are represented. Their cellular localization, the number of each representative and their name are indicated. **(B)** Schematic representation of the experimental setup and the times of sampling. **(C)** Relative abundance of the zinc transport systems. Graphs represent the correlation plots between the fold change (Log_2_ value, *y* axis) after 1 h compared to t0 (upper panel), 5 h compared to 1 h (middle panel) or 5 h compared to t0 (lower panel) of Zn treatment, and their corresponding statistical values (*x* axis). The *p*-values lower than 10^– 3^ (to the right of the vertical dotted line) were considered as statistically significant. The average values obtained for t0 (or 1 h for the middle panel) correspond to 100%. **(D)** Changes in intracellular zinc concentration after addition of 2 mM ZnCl_2_ to the medium. The metal concentrations in μg/g cells (dry weight) were determined over time in the wild type strain by ICP-MS. The concentration of Cu, as control, is also plotted on the graph.

The toxic properties of Zn are widely exploited in the context of infection control. Upon bacterial invasion, one of the host defense mechanisms is the sequestration of essential metal ions, in particular Fe, Zn, and manganese (Mn), by proteins and molecules, leading to so called “nutritional immunity” (Kehl-Fie and Skaar, 2010; Capdevila et al., 2016; Lonergan and Skaar, 2019). Conversely, during phagocytosis, toxic concentrations of Zn and copper (Cu) are delivered into the phagolysosome, participating in the destruction of the pathogen (Stafford et al., 2013; Gao et al., 2018). Thus, to successfully infect, microorganisms not only must adapt to environments of Zn deprivation or excess, but also rapidly alternate between these two extreme conditions. The opportunistic Gram-negative pathogen *Pseudomonas aeruginosa* is an ubiquitous and versatile bacterium that possesses several systems for Zn homeostasis (Figure 1A). *P. aeruginosa* is responsible for a wide array of severe infections, particularly in cystic fibrosis and immunocompromised patients (Kerr and Snelling, 2009), and Zn homeostasis systems are highly relevant to its pathogenicity (Gonzalez et al., 2019). Notably, they modulate virulence by acting on quorum sensing (Dieppois et al., 2012) as well as antibiotic resistance by repressing the expression of the route of entry for carbapenem antibiotics (Perron et al., 2004).

Numerous systems are involved in the import of Zn into *P. aeruginosa* (Pederick et al., 2015). At first, the TonB-dependent receptors ZnuD and PA2911 interact with its energizing TonB-ExbBD protein complex to translocate extracellular Zn into the periplasmic space (Gonzalez et al., 2019). Subsequently, the metal crosses the cytoplasmic membrane via either the P-type ATPase HmtA or via an ATP-binding cassette (ABC) transporter such as ZnuABC, the most common Zn uptake pathway in bacteria (Pederick et al., 2015). Revealed for the first time in *Escherichia coli*, this transporter consists of the solute-binding protein (SBP) ZnuA, which scavenges Zn in the periplasm with a high affinity and brings it to the inner membrane permease ZnuB. The energy required for this transport is provided by the cytoplasmic ATPase ZnuC (Patzer and Hantke, 1998).

Two additional ABC transporters have been described in *P. aeruginosa*: PA4063-PA4066 and PA2912-PA2914 (Pederick et al., 2015). Although these transporters have been shown to be overexpressed under conditions of Zn limitation, they remain poorly characterized and their exact contribution to Zn uptake remains to be elucidated.

The ZrmABCD system has recently been designated as a new Zn acquisition system, involving a nicotianamine-related zincophore called pseudopaline by similarity to the *Staphylococcus aureus* staphylopine (Lhospice et al., 2017; Mastropasqua et al., 2017). The molecule is synthetized by the ZrmB and ZrmC cytoplasmic enzymes and then released outside the cell by the EamA-like transporter ZrmD. Internalization of the Zn-pseudopaline complex is mediated by the TonB-dependent receptor ZrmA, in a siderophore-like manner (McFarlane and Lamb, 2017). Though many gaps remain to be filled at the mechanical level, the importance of this system under conditions of Zn limitation and its involvement in bacterial pathogenicity appear evident, especially in a cystic fibrosis context (Hermansen et al., 2018).

A considerable reservoir of Zn is represented by ribosomal proteins. Indeed, during nutrient deprivation, *P. aeruginosa* copes with metal deficiency by expressing additional ribosomal paralogous proteins, annotated as C−, which have lost their Zn finger domain (Haas et al., 2009; Gonzalez et al., 2019). A release of Zn probably results from this exchange phenomenon, that can be used by other proteins such as DNA polymerase, primase, etc. The *P. aeruginosa* PA3600-PA3601 operon encodes two C− paralogs, RpmE2 and RpmJ2, able to substitute in place and in function the ribosomal proteins RpmE and RpmJ, respectively. Recent data from *E. coli* have shown that RpmE2 and RpmJ2 are of comparable efficiency to their Zn-ribbon paralogs (Ueta et al., 2020). Such a strategy is also observed with the global transcription regulator DksA, involved in the stringent response. Under Zn-scarce conditions, the alternative protein DksA2, devoid of a Zn finger domain, is induced and takes over the functional relay from its paralog DksA (Blaby-Haas et al., 2011; Furman et al., 2013). The *dksA2* gene is found in a Zn-regulated cluster composed of approximately 10 genes including two encoding C− paralog enzymes, *pyrC2* and *folE2* (Pederick et al., 2015).

Zinc uptake and storage systems are regulated by the one-component regulator Zn uptake regulator (Zur) protein, that belongs to the ferric uptake regulatory (FUR) protein family. Zur senses the cytoplasmic concentration of the metal and its regulatory function is directly related to its ability to reversibly bind Zn (Ellison et al., 2013; Gonzalez et al., 2019). Under conditions of Zn sufficiency or excess, the regulator is found in a dimeric form containing four Zn atoms (Zur2-Zn4 conformation), which promotes DNA binding and ensures the repression of target genes by preventing RNA polymerase from initiating transcription. In *P. aeruginosa*, nine Zur binding sites were predicted, characterized by a 17-nucleotide palindromic motif overlapping the −10 of the promoter region (Pederick et al., 2015).

*Pseudomonas aeruginosa* has the ability to counter high toxic concentrations of Zn. Three families of export systems have been described in this bacterium, including the CzcCBA efflux pump, a homolog of the system found in the metal-resistant bacterium *Cupriavidus metallidurans* (Nies et al., 1989). CzcCBA belongs to the Resistance-Nodulation-Division (RND) group of the Heavy Metal Efflux (HME) family, capable of expelling excess Zn, cadmium (Cd) and cobalt (Co) from the cytoplasmic or periplasmic compartments directly outside the cell (Goldberg et al., 1999). This pump is regulated by the CzcRS two-component system (TCS), where CzcS is the transmembrane sensor and CzcR is the response regulator. CzcS detects and becomes active under conditions of excess periplasmic Zn or Cd. It then activates CzcR by phosphorylation, which in turn acts as a transcriptional activator of the efflux system, ensuring detoxification of the cell. Additionally, CzcRS TCS has been shown to activate its own transcription, promoting a positive regulation loop, but also to directly repress the OprD porin, the route of carbapenem entry, thus rendering the bacterium resistant to these antibiotics (Perron et al., 2004; Dieppois et al., 2012).

Moreover, the bacterium possesses two cation diffusion facilitators (CDF), CzcD, and YiiP. These systems act as a homodimer or heterodimer to expel Zn from the cytoplasm to the periplasm by way of a proton gradient. They also appear to be involved in maintaining membrane integrity, which would explain why mutants deleted for these CDFs are more sensitive to several antibiotics (Salusso and Raimunda, 2017).

A final layer consists of the P-type ATPase CadA, which is also responsible for detoxifying the cytoplasm by expelling Zn to the periplasm. In *P. aeruginosa*, CadA was first described as a system involved in Cd resistance (Lee et al., 2001). Similar to the *E. coli* ZntA, this protein has recently been shown to be essential for Zn resistance (Ducret et al., 2020). CadA is regulated by CadR that belongs to the MerR family of response regulators. CadR is constitutively expressed and has a high affinity for the *cadA* promoter in the apo and holo forms. The activity of this regulator is directly linked to its ability to bind Zn since it plays the dual role of repressor and activator of *cadA* transcription, depending on whether it is in conditions of Zn limitation or excess, respectively (Ducret et al., 2020).

Numerous strategies allowing Zn homeostasis have been described and characterized in *P. aeruginosa*. All these systems serve to ensure the survival of the bacterium in environments that are limited or contaminated with Zn and, interestingly, make the link between metallostasis, virulence and antibiotic resistance. Knowledge of how these systems interact with each other, however, is scarce.

Our lab previously highlighted a dynamic in the expression of Zn export systems in *P. aeruginosa*. Indeed, we had shown that the P-type ATPase CadA was not only the first line of protection against a boost in Zn, but also facilitated the induction of the major export system, the CzcCBA efflux pump (Ducret et al., 2020). These results support the hypothesis that the different systems involved in Zn resistance are not redundant, but follow a precise strategic plan, guaranteeing a rapid adaptation of the cell to variations in Zn concentrations.

The purpose of this study was to follow up on the “strategic plan” hypothesis and assess the dynamics of all the systems involved in Zn homeostasis (import/export/storage) when the bacterium transitions from starvation to surfeit, as it happens during an infection. To achieve this aim, we made use of the NanoString technology, which allows the direct counting of several mRNAs simultaneously by using digital bar-coded probe pairs (Geiss et al., 2008). We could therefore quantify transcripts of all Zn homeostasis systems, rather than following a single element of the system, and follow their dynamics over time. While we clearly observed a global import systems repression, a hierarchical induction of the export systems appeared. An additional novel partner of the Zn resistance, PA2807, a CzcE-like protein, was also discovered and characterized. Finally, the rapid induction of carbapenem resistance via OprD porin repression in the presence of Zn was precisely analyzed at the transcriptomic and proteomic levels.

## Results

In order to monitor the dynamics of Zn transport systems when *P. aeruginosa* switches from a situation of Zn starvation to Zn excess, we designed an experimental procedure described in Figure 1B. Briefly, *P. aeruginosa* PAO1 strain was cultivated at 37°C in a Zn-depleted medium (M-LB) containing 30 μM TPEN [*N*,*N*,*N*′,*N*′-tetrakis(2-pyridinylmethyl)-1,2-ethanediamine] to chelate all residual Zn for 2 h 30 min (Ellison et al., 2013). Then, 2 mM ZnCl_2_ (final concentration) was added to the culture. Previous to Zn addition (t0) and at different times afterward, cells were sampled for proteomic, transcriptomic, and Zn content analysis.

At protein levels we observed, as expected, a significant increase of all export systems during the first hour of high Zn exposure, while surprisingly the level of only two proteins involved in Zn import, ZrmB and PA1925, decreased more than 50% (Figure 1C and Supplementary Table 1). Indeed, the large majority of uptake systems significantly dropped only between 1 and 5 h after Zn addition. During this time frame, the amount of the CadA P-type ATPase decreased slightly in agreement with our previous observations on its role of early player in case of a sudden Zn excess (Ducret et al., 2020). The 5 h-period was also marked by the increasing of the CzcD protein. This very late induction took place after the CzcCBA efflux rise and may explain why this CDF was not essential for Zn resistance (Ducret et al., 2020).

When looking at the intracellular Zn concentration over time, the values changed from 0.014 to 0.14 μM in 15 min, then returned to equilibrium at a concentration of approximately 0.04 μM after 60 min (Figure 1D). It thus appears that the rapid induction of efflux systems prevails over the slow repression of import systems and it is sufficient to counteract the Zn overflow.

Finally, to decipher early events of regulation of the Zn homeostasis network, we directly monitored the transcript levels of genes involved in Zn transport using the NanoString technology (Geiss et al., 2008). Twenty-five oligonucleotide probes targeting mRNAs of the known Zn import/export/storage systems of *P. aeruginosa* were designed (Table 1). In addition, four probes targeting housekeeping genes were also designed for data normalization (Supplementary Table 2). In case of a system encoded by operon, we selected at least one gene probe to represent the whole transcription unit (e.g., *czcA* and *czcC* were chosen to monitor the expression of the *czcCBA* operon). For this analysis, RNA was extracted at t0 and 30 s, 1, 2, 5, 15, 30, and 60 min (t0.5, t1, 2, 5, 13, and 60, respectively) after Zn addition (Figure 1B). The mRNA copy number for each of the target genes was then calculated in each sample, as shown in Supplementary Table 3.

**TABLE 1:** List of the twenty-five genes monitored for zinc homeostasis analysis.

Pathway	Gene name	Gene ID	Product description
Export	*czcA*	PA2520	Cation efflux transporter
	*czcC*	PA2522	Outer membrane protein
	*cadA*	PA3690	P-type ATPase transporter
	*czcD*	PA0397	Cation diffusion facilitator transporter
	*yiiP*	PA3963	Cation diffusion facilitator transporter
Uptake	*hmtA*	PA2435	P-type ATPase transporter
	*PA1922*	PA1922	TonB-dependent receptor
	*PA2911*	PA2911	TonB-dependent receptor
	*PA2914*	PA2914	Permease of ABC transporter
	*PA4063*	PA4063	Solute-binding protein
	*PA4065*	PA4065	Permease of ABC transporter
	*PA4066*	PA4066	Solute-binding protein
	*znuA*	PA5498	Solute-binding protein
	*znuB*	PA5501	Permease of ABC transporter
	*znuD*	PA0781	TonB-dependent receptor
	*zrmA*	PA4837	TonB-dependent receptor
	*zrmD*	PA4834	Nicotianamine synthase
C+/C− paralogs	*dksA*	PA4723	Supressor protein
	*dksA2*	PA5536	Supressor protein
	*rpmE*	PA5049	Ribosomal protein L31
	*rpmE2*	PA3601	Ribosomal protein L31
	*rpmJ*	PA4242	Ribosomal protein L36
	*rpmJ2*	PA3600	Ribosomal protein L36
Others	*PA2807*	PA2807	Copper binding protein
	*oprD*	PA0958	Outer membrane porin

### Zn Export Systems

#### Dynamics of CadA and CzcCBA Expression

We recently showed that the two most important systems involved in Zn resistance in *P. aeruginosa* are the CadA P-Type ATPase and the CzcCBA RND efflux pump (Ducret et al., 2020). These two systems interact together to produce an optimal response to Zn excess, *cadA* being rapidly induced, followed by *czcCBA* expression. Afterward, *cadA* expression decreased while *czcCBA* reached its maximal expression (Ducret et al., 2020).

**FIGURE 2:**
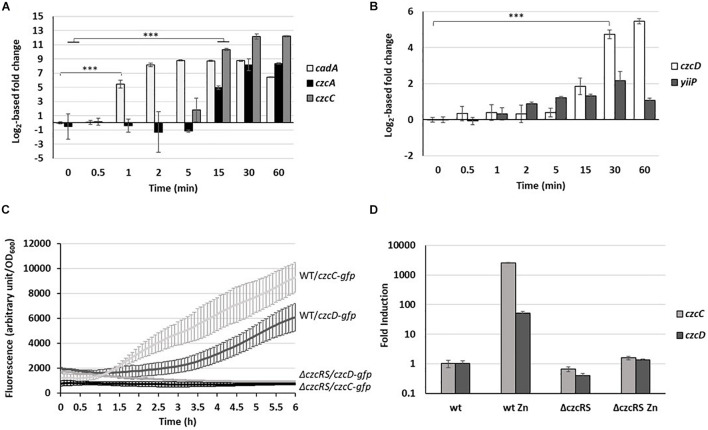
Fold change expression of export systems. **(A)** *cadA* (P-type ATPase), *czcA* and *czcC* (RND) as well as **(B)** *yiip* and *czcD* (CDF) fold change in the copy number of transcripts measured using the NanoString technology before and after addition of 2 mM ZnCl_2_ and monitored over the time. Mean values of fold change compared to t0 (before addition of Zn) and standard deviations (error bars) of three independent experiments are represented. Statistical analyses were performed according to the Student’s *t*-test and *p*-values are given as follows: ≤0.001 (^∗∗∗^). **(C)** Fluorescence measurement of *pczcC::gfp* (*czcC*) and *pczcD::gfp* (*czcD*) in the WT strain and the Δ*czcRS* mutant after addition of 2 mM ZnCl_2_. Values are normalized by optical density (OD_600_). Standard deviations (error bars) of three measurements are indicated. **(D)** qRT-PCR analysis of *czcC* and *czcD* mRNAs taken 2 h after addition of 2 mM ZnCl_2_. The amount of mRNA is represented relative to time 0 (set to 1), before addition of ZnCl_2_. Results are normalized using the *oprF* gene and standard deviations (error bars) of three independent experiments are indicated.

The *cadA* and *czcCBA* expression dynamic is also clearly visible in the NanoString experiment presented here (Figure 2A), thus confirming that this method is suitable for the fine monitoring of gene expression during the low-to-high Zn concentration transition.

After only 1 min, the amount of *cadA* mRNA rose to more than 20,000 transcripts, corresponding to a 50-fold induction (Figure 2A). The quantity of mRNA increased up to 5 min, reached a plateau, then decreased after 60 min (Figure 2A). Interestingly, a basal level of *cadA* was detected even under Zn-depleted conditions (t0) with more than 400 transcripts per 50 ng of total RNA (Supplementary Table 3). The levels of *czcA* and *czcC* transcripts (last and first gene of the *czcCBA* operon) reached their maximum after 30 min. After 1 h in the presence of the metal, a fold induction higher than 330 for *czcA* and higher than 4,700 for *czcC* was observed. Looking at the number of transcripts (Supplementary Table 3), we noticed that, unlike *czcC*, *czcA* was already present under conditions of Zn limitation. Moreover, the number of *czcA* mRNA copies detected after 1 h in the presence of the metal was twice higher than that of *czcC*. Altogether, this suggested a transcriptional independence of *czcA* that could be mediated *via* an internal transcriptional start site (iTSS) within the *czcCBA* operon (Guell et al., 2011). A similar situation has been observed in the *czc* operon of *C. metallidurans*, giving rise to different polycistronic mRNAs, some constitutively expressed and additionally inducible in a CzcR-dependent manner (Grosse et al., 2004).

#### CzcD Is Part of the CzcRS Regulon

CzcD and YiiP are two cation diffusion facilitators (CDF) that export Zn from the cytosol to the periplasm (Salusso and Raimunda, 2017). Both showed low basal expression even before Zn addition (Supplementary Table 3). However, the level of *yiip* mRNA did not increase upon Zn treatment, while *czcD* displayed a 26-fold induction, visible after 30 min (Figure 2B).

Since *czcD* is located on the *czc* locus in *C. metallidurans* (Nies et al., 1989), we wanted to determine whether *czcD* is part of the CzcRS-mediated regulation. For this purpose, we constructed a transcriptional fusion containing the *czcD* promoter controlling a *gfp* (green fluorescent protein). The expression of the fusion was tested in the wild type (WT) and Δ*czcRS* mutant strain in presence of 2 mM of ZnCl_2_. Similarly to what is observed for the *czcC* promoter (Perron et al., 2004), we could show that *czcD* expression depends on the CzcRS TCS since no fluorescence was detected after Zn induction in the Δ*czcRS* mutant (Figure 2C). This result was also confirmed by qRT-PCR (Figure 2D). The fact that *czcD* gene induction is weaker than *czcCBA* (Supplementary Table 3) might reflect its secondary role in Zn resistance (Salusso and Raimunda, 2017; Ducret et al., 2020). Altogether, these results suggest that *czcD* is part of the CzcRS regulon.

#### PA2807, a Novel Link Between Zn and Cu Homeostasis

**FIGURE 3:**
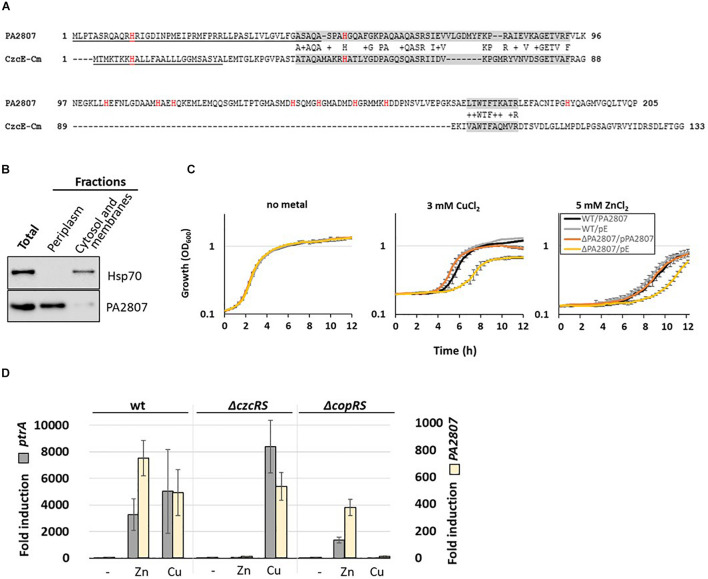
PA2807 encodes a CzcE-like protein. **(A)** Primary sequence alignment of PA2807 and CzcE from *Cupriavidus metallidurans.* The transit peptide is underscored and histidine residues are indicated in red. The two boxes of similarity are labeled in gray. **(B)** Immunoblot analysis of PA2807 localization. A culture of WT *P. aeruginosa* containing the 6His tagged version of the *PA2807* gene and its promoter on the pME6001 plasmid was induced for 2 h with 2 mM CuCl_2_. 25 μg of total protein, and an equal volume (of 25 μg of protein) of periplasmic and cytosolic-membrane fractions was separated on a 4–12% SDS PAGE. Blots were exposed to anti-6His and anti-Hsp70 (loading and cytosolic control) antibodies. **(C)** Growth curves of WT *P. aeruginosa* and the Δ*PA2807* mutant carrying the empty IPTG-inducible pMMB66EH plasmid or the pMMB66EH-*PA2807* plasmid. Strains were cultivated in LB medium in the absence or presence of 3 mM CuCl_2_ or 5 mM ZnCl_2_ with 0.1 mM IPTG. **(D)** Fold induction of *ptrA* and *PA2807* mRNA analyzed using qRT-PCR on RNA extracted after 2 h of growth in presence of 2 mM ZnCl_2_ or 2 mM CuCl_2_ as indicated. Error bars represent the standard deviations of three independent determinations.

In *C. metallidurans*, the *czc* element consists of nine genes with a *czcNICBADRSE* organization (Grosse et al., 2004). We searched for conserved amino acid sequences in *P. aeruginosa* using DIAMOND (Buchfink et al., 2015). No significant CzcI and CzcN homologs were identified. Instead, at low stringency, we found that the PA2807 protein possesses two regions of approximately 40% sequence identity to *C. metallidurans* CzcE (Figure 3A). In *C. metallidurans*, CzcE is a periplasmic protein that has been shown to bind Cu but whose expression is also induced by Zn independently of the CzcDRS regulatory element (Grosse et al., 2004; Zoropogui et al., 2008). Using SignalP 5.0 software (Nielsen, 2017), a putative signal peptide of 48 amino acids that targets PA2807 to the periplasm is predicted with a probability of 0.47 (Figure 3A). To confirm the prediction, we tested the localization of a C-terminal 6His-tagged PA2807 protein expressed *in trans* in *P. aeruginosa* and we found that it was strongly enriched in the periplasmic fraction (Figure 3B). The amino acid comparison with CzcE from *C. metallidurans* showed that PA2807 possesses an additional sequence of approximately 70 amino acids between the two conserved regions that contains 7 His residues and may constitute a metal binding site (Figure 3A).

*Pseudomonas aeruginosa* PA2807 is described as a protein of the cupredoxin family and has been shown to be involved in Cu resistance (Quintana et al., 2017). No effect on Zn resistance, however, was observed in a disk assay (Teitzel et al., 2006). To assess whether in our experimental settings the PA2807 protein is involved in Zn and/or Cu tolerance, we deleted this gene in a *P. aeruginosa* PAO1 strain and monitored the growth of the Δ*PA2807* mutant in the presence of 3 mM CuCl_2_ or 5 mM ZnCl_2_, as compared to the wild type strain (Figure 3C). The growth of Δ*PA2807* mutant was clearly delayed, indicating that the protein is indeed contributing to both Cu and Zn resistance. The mutant growth deficiency could be complemented by expressing the *PA2807* gene on a plasmid (Figure 3C). The involvement of PA2807 in Cu and Zn resistance was also confirmed on plate, using serial dilutions spotted on LB plates containing 5 mM ZnCl_2_ or 3 mM CuCl_2_ (Supplementary Figure 1).

To determine whether the transcription of *PA2807* could be induced by Zn, we measured its transcript levels via the NanoString analysis. Surprisingly, it turned out to be the most induced gene, almost 10,000 times after 1 h of induction (Supplementary Table 3). *PA2807* is part of a gene cluster comprised of *ptrA* (*PA2806*), *PA2807*, and *queF* (*PA2808*) next to the CopRS TCS (PA2809-PA2810) (Quintana et al., 2017). PtrA is a small periplasmic protein involved in Cu resistance that has been shown to be also strongly induced in the presence of Zn (Elsen et al., 2011; Lei et al., 2020). Using semi-quantitative RT-PCR, we found that the *PA2807* gene is co-transcribed as an operon with *ptrA* (Supplementary Figure 2). Interestingly, the *ptrA-PA2807* transcript expression is not only induced by CopRS in the presence of Cu, but also in a CzcRS-dependent manner in presence of Zn, as shown by qRT-PCR (Figure 3D).

Altogether, these results demonstrated that the PA2807 protein of *P. aeruginosa* shares several characteristics with *C. metallidurans* CzcE and appears to be part of the CzcRS regulon in the presence of Zn.

#### Hierarchy in the Expression of Export Systems

The induction of the expression of these four Zn export systems, namely the CadA P-type ATPase, The RND CzcCBA, the two CDFs CzcD and Yiip, along with the newly discovered CzcE homolog, PA2807, showed a precise chronology of induction, as represented in Supplementary Figure 3. Among all the export systems tested, *cadA* was the first to be induced (1 min, early induced gene). The rapid induction is probably due to the CadR regulator already bound to the *cadA* promoter region and therefore ready to transcribe *cadA* in the event of Zn excess (Ducret et al., 2020). The *czc* regulon containing the *czcCBA* efflux pump, *czcD* and *PA280*7 (*czcE*-like) induced later (15–30 min, late induced genes), while the gene encoding the CDF *yiiP* showed no induction under the conditions tested (uninduced gene).

### Zn Uptake Systems

**FIGURE 4:**
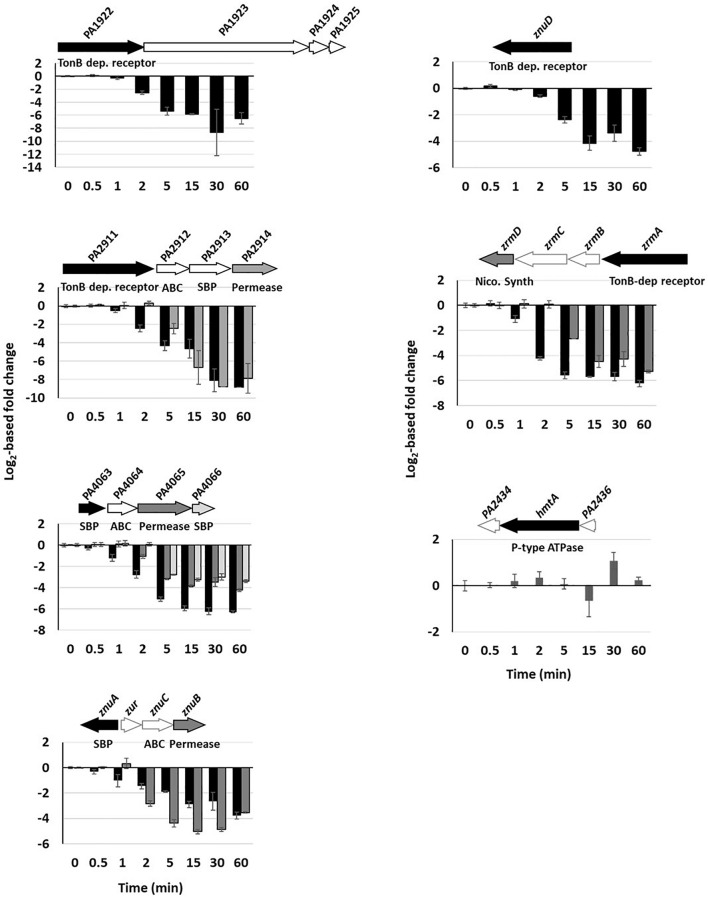
Repression of Zn import systems. The fold change of gene repressions over time of the seven import systems are shown. Fold change of the copy number of transcripts measured using the NanoString technology before and after addition of 2 mM ZnCl_2_ and monitored over time. Black, dark and clear gray in the histogram correspond to the followed gene in the genomic arrangement represented next to the graph. White arrows represent genes of the system that were not followed during this work. The fold repression gives a *p*-value ≤ 0.001 between 2 and 5 min after addition of Zn for all systems, except *hmtA* for which no repression was detected.

Unlike the protein levels (Figure 1C), the mRNA levels of Zn import systems were strongly and rapidly repressed once Zn was added to the medium (Supplementary Figure 3 and Supplementary Table 3). Contrarily to export systems, however, no hierarchy in the regulation was evident and the decrease in the mRNA abundance of all uptake systems was statistically relevant (*p*-value ≤ 0.001) between 2 and 5 min after Zn addition (Figure 4). One exception to this reduction was observed with the gene encoding the P-type ATPase HmtA, whose mRNA level remained constantly low (Supplementary Table 3). This lack of repression could be due to the fact that this system is mainly involved in Cu import (Lewinson et al., 2009). Finally, among the three ABC transporters involved in Zn uptake, the PA4063-4066 system showed the highest expression (Supplementary Table 3).

### C− and C+ Paralog Proteins

**FIGURE 5:**
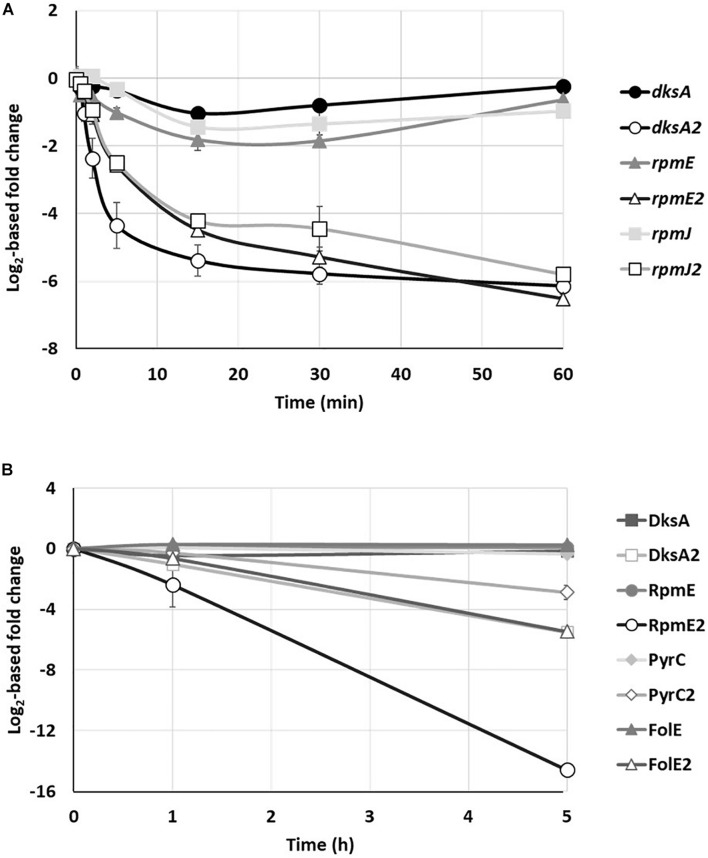
Kinetics of C+/C– protein family. **(A)** Fold change of *rpmE*, *rpmJ* and *dksA* transcripts and their C– paralogs over time, after addition of 2 mM ZnCl_2_. **(B)** Fold changes in the amount of DksA, RpmE, PyrC, and FolE proteins and their C– paralog after addition of 2 mM ZnCl_2_. Mean values of fold change compared to t0 (before addition of Zn) and standard deviations (error bars) of three independent experiments are represented.

Under conditions of Zn deficiency, the stringent response regulator DksA is replaced by its DksA2 ortholog, which is devoid of a Zn finger domain (C− form) (Blaby-Haas et al., 2011). When Zn reaches sufficient cytoplasmic concentration, *dksA2* transcription is switched off by Zur and DksA takes over its place and function. In agreement with this, a rapid drop in the level of *dksA2* mRNA was observed: after 1 min of metal exposure only half of the number of mRNAs remained (Figure 5A and Supplementary Table 3). A similar repression profile was shown for the two ribosomal genes *rpmE2* and *rpmJ*2 encoding Zn deficient orthologs of RpmE and RpmJ, respectively. Unlike their C− substitutes, the mRNA levels of *dksA*, *rpmE*, and *rpmJ* remain constant, indicating the simultaneous expression of the three orthologous genes when the environment is depleted of metal. Are both C+ and C− proteins expressed at the same time or an additional regulatory mechanism occur at the next levels of gene expression? To address this question, we looked at the proteomic analysis focusing on the DksA and RpmE proteins (RpmJ was not detected) and their C− orthologs (Figure 5B and Supplementary Table 2). Intriguingly we observed that, while RpmE2 and DksA2 disappeared rapidly when Zn is added in excess, the proteins RpmE and DksA remain present, even in the absence of Zn. A similar regulation is also observed with the two zur-regulated enzymes (Pederick et al., 2015), FolE (GTP cyclohydrolase) and PyrC (dihydroorotase) whose concentrations remain constant in the absence and presence of Zn, while their C− orthologs, FolE2 and PyrC2 decrease as soon as excess Zn is added to the medium (Figure 5B).

### Zur Affinity

The transcriptional repression of Zn import systems as well as the genes encoding C− proteins are mediated by Zur. A Zur box was predicted for each of the repressed genes or operons (Pederick et al., 2015) and Zur was shown to directly bind to the *znuA* and *dksA2* promoters (Blaby-Haas et al., 2011; Ellison et al., 2013). In *P. aeruginosa* this repressor is characterized by 2 Zn binding sites, the structural site located in the C-terminal region and a regulatory M-site (Ellison et al., 2013). The Zur dimer contains 2 Zn^2+^ ions at the structural site and depending on the cytoplasmic Zn concentration, 0, 1, or 2 Zn^2+^ ions at the regulatory M-site. In *Bacillus subtilis*, Zur promoter affinity depends on the amount of Zn linked to the regulatory site of the protein, allowing a fine cellular response to Zn by precisely tuning Zur action (Ma et al., 2011).

**FIGURE 6:**
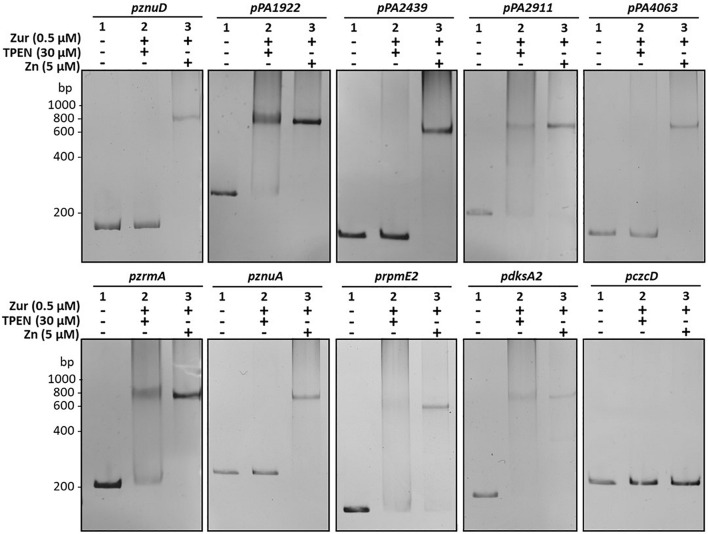
Zur binds to the diverse *zur* box with different affinities. Electrophoretic mobility shift assays using purified Zur protein and the different target promoters. The *czcD* promoter (*pczcD*) was used as a negative control. 30 ng of DNA were mixed with (1) 0 nM of Zur, 5 μM ZnCl_2_; (2) 500 nM Zur and 30 μM TPEN (Zn depleted condition); and (3) 500 nM Zur and 5 μM ZnCl_2_, as indicated. Reactions were loaded on non-denaturing 7.5% polyacrylamide gels, stained with ethidium bromide and viewed under UV light.

No clear hierarchy in the repression of Zur regulated genes could be observed with the NanoString experiment after addition of 2 mM ZnCl_2_ (Supplementary Figure 3 and Supplementary Table 3). We therefore used a more refined analysis to investigate a possible hierarchy in the affinity of the Zur regulator for the various putative Zur targets in *P. aeruginosa*. To this aim, we purified Zur (Supplementary Figure 4) and carried out Electrophoretic Mobility Shift Assay (EMSA) assays, in the absence (30 μM TPEN) or presence of Zn (5 μM ZnCl_2_) (Figure 6).

All the promoters tested were shifted, including the promoter of the PA2439 gene (first gene of the locus containing *hmtA*), for which a weak putative Zur binding site had previously been determined *in silico* (Pederick et al., 2015). Interestingly, two distinct target profiles were emerging from the EMSA analysis. One set of targets exhibited a complete DNA-Zur interaction only in the presence of Zn (*znuA*, *znuD*, *PA4063, PA2439*, and *rpmE2* promoters), while the other showed binding even in the absence of metal. This latter case represented the promoters of the *PA1922*, *PA2911*, and *zrmA* genes, which encode for TonB-dependent receptor components (Pederick et al., 2015) and the *dksA2* gene, involved in stringent response under Zn starvation conditions (Blaby-Haas et al., 2011).

#### Induction of Carbapenem Resistance

**FIGURE 7:**
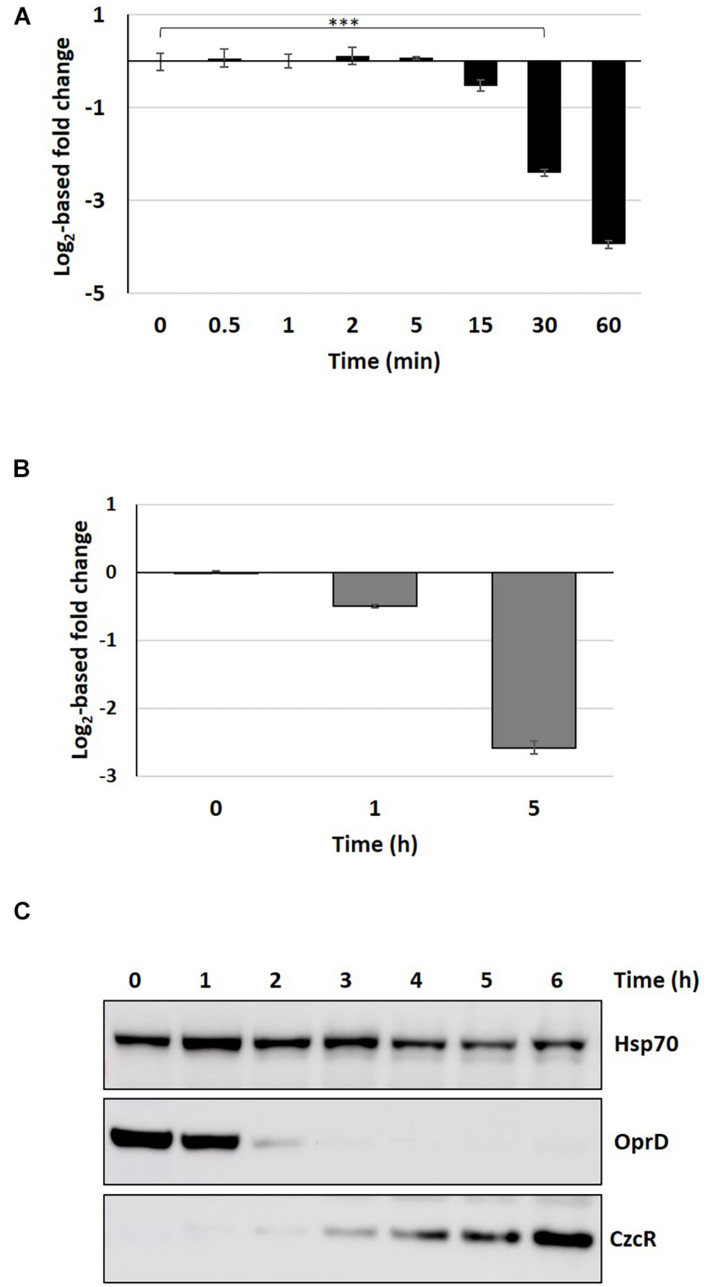
Proteomic and transcriptomic kinetics of OprD repression. **(A)** Fold change in *oprD* mRNA obtained by the NanoString technique after addition of 2 mM ZnCl_2_. Mean values of fold change compared to t0 (before addition of Zn) and standard deviations (error bars) of three independent experiments are represented. Statistical analyses were performed according to the Student’s *t*-test and *p*-values are given as follows: ≤0.001 (***). **(B)** Fold change in OprD porin compared to t0 (before addition of Zn), 1 and 5 h after addition of 2 mM Zn. **(C)** Western blot analysis of total protein sampled without Zn (t0) and after 1–6 h following the addition of 2 mM ZnCl_2_. Blots were decorated with anti-OprD, anti-CzcR, and anti-Hsp70 as loading control.

The response to Zn in *P. aeruginosa* is linked to carbapenem antibiotic resistance. The route of entry of these antibiotics into the cell is the OprD porin, whose expression is repressed in the presence of Zn at both transcriptional and post-transcriptional level via the CzcRS TCS and the Hfq RNA chaperone, respectively (Perron et al., 2004; Ducret et al., 2016). Recently, we found that the metal concentrations present in phagolysosomes are sufficient to induce the Zn response and therefore carbapenem resistance in *P. aeruginosa* (Ducret et al., 2020). We then followed the kinetics of carbapenem resistance induction by quantifying *oprD* downregulation at the mRNA and protein levels (Figure 7). A delay of 30 min was sufficient to observe a significant drop in *oprD* mRNA levels (Figure 7A) and the protein was only slightly present (less than 20%) after 5 h of induction (Figure 7B and Supplementary Table 1). This drop was also confirmed at protein levels by a Western blot experiment, where the protein was no longer detectable after 3 h of metal addition (Figure 7C).

## Discussion

**FIGURE 8:**
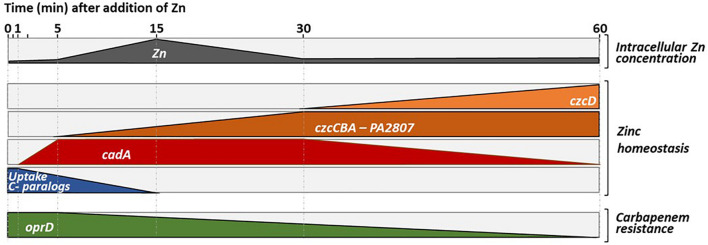
Representation of the dynamics of Zn homeostasis in *Pseudomonas aeruginosa.* The first block represents the intracellular Zn concentration, which increases when the cells move from a Zn-poor to a Zn-rich environment. An increase in intracellular Zn concentration is observed for up to 15 min, the time it takes for the import systems to be repressed and for the export system to start to eliminate the excess Zn (see second block and discussion). Later it appears that the ATP-consuming CadA system, is replaced by a less energy-demanding CzcD system that takes over to pump cytoplasmic Zn into the periplasm. This representation, highlights the repression of the expression of the OprD porin whose abundance is inversely proportional to resistance to the family of carbapenem antibiotics (last block).

To infect and take advantage of its host, a pathogen must be able to adapt quickly to extreme conditions, including rapid changes in Zn concentration. *P. aeruginosa* is typically a bacterium armed to counter this kind of situations, as shown by the numerous systems involved in Zn homeostasis. These systems, together with the ability to express them in a timely fashion, contribute to *P. aeruginosa* ability to infect hosts (Gonzalez et al., 2019). In this study, we carried out a systematic investigation of the expression dynamics of the entire *P. aeruginosa* Zn homeostasis network when switching from a Zn depleted environment to a Zn excess situation, a transition mimicking what happens during an infectious process (Djoko et al., 2015). Our analysis allowed us to draw a global model of Zn homeostasis gene expression organization in *P aeruginosa*, as illustrated in Figure 8.

As a first reaction, we observed a quick induction of *cadA* expression (after 1 min). Presumably, this serves to rapidly evacuate the Zn excess out of the cytoplasm into the periplasm (Ducret et al., 2020). Downregulation of transcripts for uptake systems is also occurring fast, probably to prevent new Zn ions from entering the cell. Surprisingly, at the protein level this repression is visible only after 5 h, suggesting a slow turnover of Zn uptake proteins. Given the importance of Zn for survival, this might be the result of a “prudential regulatory strategy,” to assure a prompt Zn uptake in case of the metal concentration decreases suddenly.

In a second step, the CzcCBA major efflux system is induced to detoxify all the cellular compartments, thus allowing the bacterium to thrive in an environment highly contaminated with Zn. In agreement with this, a decrease in total cellular Zn is observed as the expression of *czcCBA* increases (Figure 1D). The CzcD CDF is not an essential component in metal resistance (Ducret et al., 2020), but we show here to be part of the *czcRS* regulon (Figure 2) and therefore responding to the periplasmic Zn concentration. Considering its late induction, CzcD may contribute to the maintenance of a steady state level of Zn resistance by continuing to pump Zn into the periplasm and allowing the CzcS to remain active while *cadA* expression decreases. One may consider that the P-type ATPase system CadA, despite being very efficient, uses ATP as an energy source while CzcD is driven by proton-motive forces and, therefore, might be more profitable for the cell in terms of energy optimization.

The *PA2807* gene was previously shown to be induced by the CopRS system in the presence of Cu (Quintana et al., 2017). Here, we show that it encodes a CzcE-like protein involved in Zn resistance and also induced by the CzcRS systems in the presence of Zn. The role of this periplasmic protein in Zn and Cu resistance requires further investigation, but we could confirm the link between Zn and Cu resistance that has already been observed in other bacteria. For instance, in *Pseudomonas stutzeri*, a common overlapping response to Cu and Zn by common regulator DNA binding motifs has been elegantly demonstrated (Garber et al., 2018). In *P. aeruginosa*, the induction of CopRS induces the expression of the CzcRS TCS (Caille et al., 2007). In general, both Zn and Cu resistance may allow bacteria to resist to the toxic boost of metals that are discharged into the phagosome of macrophages to kill the pathogens (Botella et al., 2012; Djoko et al., 2015). Shift assays have shown a greater affinity of Zur for promoters controlling the expression of *PA1922*, *PA2911, zrmA*, and *dksA2* genes. Interestingly, all these genes are also involved in cobalt (Co) homeostasis, since *PA1922* is located within an operon that contains a *cobN*-like gene (*PA1923*), *PA2911* is co-transcribed with *PA2914*, a homolog of the cobalamin ABC permease (Pederick et al., 2015) and pseudopaline, involving the *zrm* system, has been shown to transport metals such as Zn, Co, Fe, and nickel (Ni) (Zhang et al., 2019). Similarly, *dksA2* is also part of an operon including *PA5535*, a *cobW*-like gene, involved in cobalamin biosynthesis. These results might reveal a putative Co-dependent regulatory activity of Zur, which could be linked with vitamin B12 (cobalamin) synthesis (Osman et al., 2021). Our *in vitro* conditions, by creating a Zn deficiency using TPEN, could displace the filling of Zur with Co (Osman et al., 2017). Some promoters would thus also appear to react first to a Co boost. The analysis of the sequences of the different Zur boxes did not allow us to highlight any particular signature. The DNA-Zur-Co interaction would deserve further investigation.

In *E. coli*, each of the 50,000 ribosomes present in exponentially growing cell contains about 3 Zn^2+^ ions, resulting in the recruitment of 75% of all the intracellular Zn available (Blaby-Haas et al., 2011). Under conditions of Zn deficiency, the ribosomal proteins RpmE and RpmJ, along with the transcription factor DksA and the two enzymes FolE and PyrC all of which contain Zn-ribbon motifs (and are thus called C+) are replaced by their C− paralog proteins (RpmE2, RpmJ2 DksA2, FolE2, and PyrC2), which lack the key Cys residues and do not require Zn for their function. This replacement frees Zn, which becomes available for other essential biological tasks (Gabriel and Helmann, 2009). A similar mechanism is found also in *B. subtilis*, in which the RpmE protein is degraded under Zn depleted conditions (Akanuma et al., 2006). As expected we found a rapid drop of C− proteins upon Zn excess in *P. aeruginosa*. However, unlike *B. subtilis*, *P. aeruginosa* C+ proteins are constitutively present even in conditions of Zn deficiency (t0). It is possible that the presence of these proteins reflects a regulatory strategy that guarantees a pool of proteins rapidly functional as soon as Zn is available.

In summary, this work shows for the first time the dynamics of the components of the Zn homeostasis network in *P. aeruginosa.* The use of the NanoString technology allowed us to precisely quantify several transcripts simultaneously and to follow their dynamics over time. The complementary proteomic analyses allowed us to observe the final outcome of this regulation and to visualize the Zn homeostasis systems at steady state (i.e., after 5 h of growth in presence of Zn excess). Our findings are important to identify the molecular mechanisms favoring host colonization and infection, as well as to understand the environmental signals leading to the insurgence of antibiotic resistance. Specifically, the CzcRS-dependent repression of the *oprD* porin renders *P. aeruginosa* resistant to carbapenem in presence of Zn (Perron et al., 2004). According to our data, the OprD protein cannot be detected by western blot after 3 h of Zn treatment (Figure 7C). We have recently reported OrpD repression during phagocytosis (Ducret et al., 2020). Macrophages, and in general the immune system response, could therefore represent an important underestimated cause of carbapenem phenotypic resistance. Understanding the molecular mechanism favoring the appearance of carbapenem resistance will be thus fundamental to optimize the use of these last resort antibiotics.

## Materials and Methods

### Bacterial Strains and Culture Media

The bacterial strains and plasmids used for this study are listed in Supplementary Table 4. Cultures requiring initial Zn deficiency conditions were carried out in modified Luria-Bertani medium (AppliChem) supplemented with 30 μM of *N*,*N*,*N*′,*N*′-tetrakis(2-pyridinylmethyl)-1,2-ethanediamine (TPEN, Biotum), as described previously (Ducret et al., 2020). Otherwise standard Luria Broth medium (AppliChem) was used. Cultures were incubated at 37°C and when required, antibiotics were added to the medium at the following concentrations: 200 μg/mL carbenicillin (Cb, phytotechlab), 50 μg/mL Gentamycin (Gm, AppliChem) and 50 μg/mL tetracycline (Tc, Axxora) for *P. aeruginosa* or 100 μg/mL ampicillin (Ap, AppliChem) and 15 μg/mL Tc or Gm for *E. coli*.

### Genetic Manipulations

DNA cloning was performed according to standard procedures (Sambrook and Russell, 2001). For Polymerase Chain Reactions, *P. aeruginosa* genomic DNA was used as template and the primers are listed in Supplementary Table 5. Restriction and ligation enzymes (Promega) were employed according to the manufacturer’s instructions. Resulting recombinant plasmids were inserted into *E. coli* DH5α and confirmed by sequencing prior to being transformed into the *P. aeruginosa* wild type or the indicated mutant strains by electroporation (Choi et al., 2006).

Mutants were created according to the following strategy: two fragments flanking either the *PA2807* or the *copRS* TCS locus were amplified by PCR, digested with EcoRI/KpnI for the first insert and KpnI/BamHI for the second insert and ligated into the corresponding sites of the pME3087 suicide vector (Voisard et al., 1994). After sequencing verification, recombinant plasmids were transformed into *P. aeruginosa* wild type strain. Merodiploids were resolved as previously mentioned (Ye et al., 1995) and the specific chromosomal deletions were confirmed by PCR amplification and sequencing.

For complementation of the Δ*PA2807* mutant, the full *PA2807* gene was amplified by PCR, digested with EcoRI and BamHI restriction enzymes and cloned into the corresponding sites of the IPTG-inducible pMMB66EH vector (Furste et al., 1986). The *PA2807* gene was also cloned with a C-terminal 6His tag into the pME6001 promoter-less vector (Blumer et al., 1999). To this aim, the full gene and its promoter region were obtained by PCR and inserted into the pME6001 plasmid between the BamHI and HindIII restriction sites. For *czcD::gfp* fusion, the *czcD* promoter was amplified by PCR, digested with the KpnI and BglII enzymes and then ligated in the corresponding sites of the pBRR1-*gfp* plasmid (Ouahrani-Bettache et al., 1999).

### RNA Extraction

Total RNA was isolated from the *P. aeruginosa* wild type or mutant strains as formerly explained (Ducret et al., 2020). Briefly, overnight cultures were diluted to an optical density at 600 nm (OD_600_) of 0.1 and grown for 2 h 30 min in M-LB containing 30 μM TPEN. 0.5 mL of each culture was mixed with 1 mL of RNA Bacteria Protect Reagent (Qiagen) immediately after 2 mM ZnCl_2_ induction (t0) and after several time points as indicated in the different figures. Total RNAs were extracted with an RNeasy mini kit (Qiagen) according to the manufacturer’s instructions. 5 μg of total RNA were treated with RQ1 RNase-free DNAse (Promega) for 2 h at 37°C, followed by phenol/chloroform extraction and ethanol precipitation.

### NanoString nCounter Expression Analysis

mRNA content was analyzed at the iGE3 Genomics Platform (Faculty of Medicine, University of Geneva). 50 ng of total RNA were hybridized with multiplexed NanoString probes listed in Supplementary Table 2 and samples were processed according to the published procedure (Geiss et al., 2008). Barcodes were counted for 490 fields of view per sample. Background correction was performed by subtracting the mean +2 standard deviations of the negative controls for each sample. Values < 1 were set to 1. Positive controls were used as quality assessment: the ratio between the highest and the lowest average of the positive controls among samples was below 3. Counts for target genes were then normalized with the geometric mean of the four reference targets: *fbp, ppiD, and rpoD*, selected according to Gifford et al. (2016) and *oprF*, usually used in the lab to normalize RT-qPCR. The stability of these genes has been verified using the geNorm algorithm (Vandesompele et al., 2002).

### Quantitative RT-PCR Analysis

500 ng of total RNA was reverse-transcribed using random hexamer primers (Promega) and Improm-II reverse transcriptase (Promega) according to the supplier’s instructions. The reverse transcriptase was then heat-inactivated and the resulting cDNAs were diluted tenfold in water. Quantitative PCR was performed in technical duplicates, using SYBR Select Master Mix (Applied biosystem), according to the supplier’s instructions. Results were analyzed as previously described (Schmittgen and Livak, 2008) and normalized with the *oprF* gene.

### Semi-Quantitative PCR Analysis

500 ng of total RNA from WT *P. aeruginosa* grown in LB, LB supplemented with 2 mM ZnCl_2_ or with 2 mM CuCl_2_ was reverse-transcribed as described above and diluted tenfold in water. 28 cycles of PCR amplification were performed on the cDNAs and on the corresponding RNA dilution (negative control) as well as on genomic DNA (positive control) using the primers described in Supplementary Table 5. Amplicons were analyzed on a 2% agarose gel stained with ethidium bromide using a standard procedure.

### GFP-Reporter Fusions

Green fluorescent protein fusion assays were carried out as previously described (Ducret et al., 2020). Precultures of wild type and Δ*czcRS* strains carrying the *pczcC::gfp* or *pczcD::gfp* constructions were diluted to an OD_600_ of 0.1, transferred to a 96-well black plate (Costar) and grown for 2.5 h with shaking before being induced with 2 mM ZnCl_2_ (t0). Fluorescence at 528 nm was measured every 15 min using a Microplate reader (Synergy HT, BioTek instrument) and normalized with the OD_600_ values.

### Growth Tests

Growth experiments were undertaken to investigate the metal susceptibility of the Δ*PA2807* mutant strain compared to the wild type, whether complemented or not with the *PA2807* gene. Overnight cultures of the different strains were diluted to an OD_600_ of 0.05 in LB medium containing 200 μg/mL carbenicillin and 0.1 mM IPTG (isopropyl β-D-1-thiogalactopyranoside). For growth curve analysis (Figure 3C), cultures were supplemented or not with either 3 mM CuCl_2_, or 5 mM ZnCl_2_, transferred to 96-well plates (Costar) and incubated at 37°C with shaking. Absorbance at 600 nm was measured every 15 min using a Microplate reader (Synergy HT, BioTek instrument). For growth spot assays (Supplementary Figure 1), cultures were grown for 2 h in LB containing 200 μg/mL carbenicillin and 0.1 mM isopropyl β-D-1-thiogalactopyranoside (IPTG; Axon Lab), then diluted to 10^–7^. 10 μl of each dilution was spotted onto LB plates, supplemented or not with either 3 mM CuCl_2_ or 5 mM ZnCl_2_ and incubated at 37°C for 24 h.

### Western Blot Analyses

For PA2807 cellular localization, a fractionation procedure was adapted from Elsen et al. (2011). For this purpose, an overnight culture of a WT strain containing the 6His-tagged PA2807 version was diluted to an OD_600_ of 0.1 in LB supplemented with 50 μg/mL gentamycin and 2 mM CuCl_2_. After 5 h of growth, 1 mL of cells was harvested and total proteins were solubilized at 2 mg/mL in 2X SDS-gel sample buffer (Sambrook and Russell, 2001) (an OD_600_ of 1 gives 0.175 mg/mL of protein). In parallel, 15 mL of culture were washed once with TMP buffer (10 mM Tris–HCl, 200 mM MgCl_2_, 1 mM AEBSF, pH8). Cells were then resuspended in 1 mL TMP buffer containing 0.5 mg/mL lysozyme (Fluka) and incubated at room temperature for 30 min before spinning down. The supernatant corresponds to the periplasmic fraction. The pellet, corresponding to membrane plus cytosolic fractions, was resuspended in the same volume of TMP buffer and sonicated. Both fractions were mixed with an equal volume of 2X SDS-gel sample buffer.

In order to investigate the kinetics of OprD protein repression over the time, an overnight WT *P. aeruginosa* culture was diluted to an OD_600_ of 0.05 in M-LB supplemented with 30 μM TPEN and incubated for 2 h 30 min at 37°C. 1 mL of culture was collected and centrifuged immediately prior to being induced with 2 mM ZnCl_2_ and each hour as indicated in Figure 7C. All pellets were solubilized in the appropriate volume of 2X SDS-gel sample buffer to a total protein concentration of 2 mg/mL (an OD_600_ of 1 gives 0.175 mg/mL protein). Proteins were separated on SDS PAGE, using 4–12% precast gels (Invitrogen). Membrane transfer was performed with an iBlot 2 transfer stack (Invitrogen), according to the manufacturer’s instructions. Nitrocellulose membrane was incubated with anti-OprD or anti-penta-His and anti-Hsp70 antibodies as previously described (Dieppois et al., 2012). Blots were revealed by chemiluminescence (SuperSignal, Thermo Fisher Scientific), using the Amersham Imager 680 System.

### Intracellular Zn Concentration

The procedure was adapted from D’Orazio et al. (2015). An overnight culture was diluted to an OD_600_ of 0.1 in M-LB supplemented with 30 μM TPEN and incubated 2 h 30 min at 37°C. 1 mL of cells were collected and washed once with Phosphate Buffered Saline (PBS, Gibco) containing 1 mM EDTA (Promega). The culture was then induced with 2 mM ZnCl_2_ and a 1 mL-sample was collected at 5, 15, 30, and 60 min after Zn addition and processed in the same manner, to remove any traces of Zn. Pellets from the whole kinetic sequence were deep-frozen, freeze-dried, and kept in the dark pending analyses. Total dry material was transferred into polypropylene (PP) tubes to perform acid digestion. This was done for 3 h at 90°C placing the PP tubes on Teflon heating blocks after adding 2 mL hydrochloric acid and 1 mL nitric acid (10 M HCl and 14 M HNO_3_, respectively, both Suprapur, Merck^®^) following protocol adapted from Abdou et al. (2020) for trace metal quantification in biological matrices. Cooled digestates were then diluted in 10 mL MilliQ^®^ water and centrifuged at 4,000 rpm for 10 min (20°C). The supernatant was stored in acid-cleaned PP tubes. Total Zn concentrations were quantified by Inductively Coupled Plasma-Mass Spectrometry (ICP-MS, 7900 Agilent^®^) in samples diluted 10 fold with 1 % HNO_3_. Copper (Cu) concentrations were also quantified. Since no Certified Reference Material (CRM) exist for trace metal content in bacteria; other certified biological matrices were analyzed, consisting in plankton material and seaweed (BCR^®^-414 and CD^®^200, respectively). Their analyses provided satisfactory results with recoveries for both Zn and Cu concentrations >90% and precision of ∼ 10% (*n* = 6). At least triplicate of each condition was analyzed (vertical error bars in the graphics). Detection limits (3 × blank standard deviation) was estimated to 1.5 μg/g and 0.18 μg/g for Zn and Cu, respectively, according to an OD_600_ of 1 resulting in 0.39 g L^–1^ of cell dry weight (Glazyrina et al., 2010).

### Zur Expression and Purification

The *zur* gene was amplified by PCR, cut with BamHI and EcoRI restriction enzymes and cloned into the pGEX-2T vector. The resulting plasmid was then transformed into the *E. coli* BL21 strain. Induction and purification were performed as described previously for the CadR protein (Ducret et al., 2020). After removal of the GST tag with the thrombin protease, the protein was dialyzed-concentrated against PBS containing 1 mM dithiothreitol (DTT) and 50% glycerol. Protein purity was checked on SDS-Page (Bio-Rad) stained with Coomassie blue (Supplementary Figure 4) and stored at −70°C until use.

### Electrophoretic Mobility Shift Assay (EMSA)

For the EMSA experiments, all DNA promoters indicated in Figure 6 were obtained by PCR and purified on agarose gel. Binding assays were performed according to the procedure already described (Ducret et al., 2020). Briefly, reactions were performed with a mixture composed of the 5X Zn-less Binding Buffer (50 mM Tris, 200 mM KCl, 50 mM MgCl_2_, 5 mM DTT, and 25% Glycerol), 30 ng of DNA, a Zn excess (5 μM) or deficiency (30 μM TPEN) in presence or absence of 500 nM Zur protein and incubated at room temperature for 30 min. Samples were then separated at 4°C on a 7.5% polyacrylamide native gel containing 2.5% glycerol in Tris Borate Buffer. Binding capacity was analyzed by staining the gel with 0.1% ethidium bromide and revealed with UV light using a NuGenius instrument.

### Proteomic Analyses

Expression of the DksA and RpmE C−, C+, and OprD proteins was investigated by label-free proteomic analysis. Briefly, an overnight culture of the WT *P. aeruginosa* strain was diluted to an OD_600_ of 0.1 in M-LB supplemented with 30 μM TPEN and incubated for 2 h 30 min at 37°C. 1 mL of culture was collected immediately before (t0) and after 1 and 5 h induction with 2 mM ZnCl_2_. A culture pellet was resuspended in an appropriate volume of 50 mM NH_4_CO_3_ buffer in order to adjust the concentration to 0.5 mg/mL total proteins. Cells were digested with 0.1% RapiGest SF Surfactant (Waters) for 30 min at 60°C followed by 5 min at 100°C.

Extract contents were analyzed by ElectroSpray Ionization-Liquid Chromatography–Mass/Mass Spectrometric (ESI-LC-MS/MS) at the Proteomics Core Facility (Faculty of Medicine, University of Geneva). Raw data were processed using Proteome Discoverer 2.3 Software. Label-free quantification was performed using the “Top 3 precursor Intensity” method. Normalization was applied as well as an ANOVA analysis of variance with Bonferroni multiple test correction. A minimum of 10^5^, corresponding to the minimum detection value, was fixed for targets with zero values.

### Experimental Relevance and Statistical Data

All experiments were performed, at least, in triplicate. For tables and graph representations, mean values or fold changes are shown in the figures, along with the standard deviations. When indicated, statistical analysis was performed according the Student’s *t*-test and significance *p*-value was set to *p* ≤ 0.001 (^∗∗∗^). For others, the figures show an indicative experiment.

## Data Availability Statement

The data presented in the study are deposited in the Gene Expression Omnibus (GEO) repository, accession number GSE183060.

## Author Contributions

VD, MV, and KP contributed to conception and design of the study, and wrote the manuscript. All authors contributed to formal analysis, investigation, manuscript revision, read, and approved the submitted version.

## Conflict of Interest

The authors declare that the research was conducted in the absence of any commercial or financial relationships that could be construed as a potential conflict of interest.

## Publisher’s Note

All claims expressed in this article are solely those of the authors and do not necessarily represent those of their affiliated organizations, or those of the publisher, the editors and the reviewers. Any product that may be evaluated in this article, or claim that may be made by its manufacturer, is not guaranteed or endorsed by the publisher.

## Supplementary Material

Supplementary Figure 1 PA2807 and metal resistance. Spot test assay of a serial dilution of the WT and the Δ*PA2807* mutant carrying either an empty pMMB66EH plasmid (pE) or the *PA2807* gene cloned under the inducible *tac* promoter of the pMMB66EH plasmid (pPA2807). After induction with 0.1 mM IPTG 10 μL of the various dilutions were spotted onto LB, LB + 3 mM CuCl_2_ or 5 mM ZnCl_2_, as indicated and incubated for 24 h at 37°C.

Supplementary Figure 2 Co-transcription of the *ptrA* and *PA2807 genes*. Semi-quantitative RT-PCR analysis of the three amplicons (indicated in the upper panel) on RNA isolated from *P. aeruginosa* grown in LB, LB containing 2 mM ZnCl_2_ or 2 mM CuCl_2_. Primers used for each amplicon are indicated in the Supplementary Table 2. The amplification products were analyzed on a 2% agarose gel stained with ethidium bromide (lower panel). RNA corresponds to the negative control (without reverse transcriptase) and the Genomic DNA is used as positive control.

Supplementary Figure 3 Transcription dynamics of genes involved in zinc homeostasis. mRNA counts of export **(A)** and import **(B)** systems as determined by NanoString analysis.

Supplementary Figure 4 Purification of the Zur Protein. 5 μg of purified Zur protein were loaded onto a 4–12% SDS-PAGE and stained with Coomassie Blue.

Supplementary Table 1 Proteomic results for systems involved in Zn homeostasis before and after addition of 2 mM ZnCl_2_, as indicated. Mean values of three independent experiments and standard deviations are indicated. Undetected proteins are highlighted in gray. Only one value of the triplicate was obtained for FolE.

Supplementary Table 2 NanoString codeset details.

Supplementary Table 3 mRNA counts of the target genes involved in zinc homeostasis over time, after addition of 2 mM ZnCl_2_. Mean values and standard deviations of three independent experiments are represented. The software assigned an arbitrary value of 1 to undetected mRNAs (gray boxes).

Supplementary Table 4 Strains and plasmids used in this study.

Supplementary Table 5 Primers used in this study.
