# Supplementary material for: Global Analysis of the Zinc Homeostasis Network in Pseudomonas aeruginosa and Its Gene Expression Dynamics
Source: Front Microbiol. 2021 Oct 8;12:739988. doi: 10.3389/fmicb.2021.739988 (PMC8531726; doi:10.3389/fmicb.2021.739988)
Supplement: Supplementary Figure 1 — PA2807 and metal resistance. Spot test assay of a serial dilution of the WT and the ΔPA2807 mutant carrying either an empty pMMB66EH plasmid (pE) or the PA2807 gene cloned under the inducible tac promoter of the pMMB66EH plasmid (pPA2807). After induction with 0.1 mM IPTG 10 μL of the various dilutions were spotted onto LB, LB + 3 mM CuCl2 or 5 mM ZnCl2, as indicated and incubated for 24 h at 37°C. [file Data_Sheet_1.zip › Data Sheet 1/Table 1.PDF]

Table S1: Proteomic results for systems involved in Zn homeostasis before and after addition of 2 mM $ZnCl_2$, as indicated. Mean values of three independent experiments and standard deviations are indicated. Undetected proteins are highlighted in grey. Only one value of the triplicate was obtained for FolE.

			Average of AUC (Area Under the Curve)								
Pathway	Protein	ID	t0			1h			5h		
Export	CzcA	PA2520	1.00E+05	+/-	0.00E+00	3.62E+08	+/-	4.71E+07	1.47E+09	+/-	4.51E+07
	CzcB	PA2521	1.00E+05	+/-	0.00E+00	7.33E+08	+/-	2.36E+07	2.79E+09	+/-	9.50E+07
	CzcC	PA2522	1.00E+05	+/-	0.00E+00	3.94E+08	+/-	3.60E+07	1.64E+09	+/-	9.29E+07
	CadA	PA3690	1.93E+07	+/-	4.11E+06	1.18E+09	+/-	2.89E+07	4.42E+08	+/-	2.78E+07
	CzcD	PA0397	1.00E+05	+/-	0.00E+00	6.67E+04	+/-	5.77E+04	2.38E+08	+/-	8.02E+06
	YiiP	PA3963									
Uptake	HmtA	PA2435	1.45E+07	+/-	6.86E+06	8.13E+06	+/-	0.00E+00	1.00E+05	+/-	0.00E+00
	PA1922	PA1922	2.27E+08	+/-	5.29E+06	1.49E+08	+/-	7.21E+06	1.64E+07	+/-	2.00E+06
	PA1923	PA1923	1.73E+08	+/-	3.92E+07	3.38E+07	+/-	7.53E+06	1.00E+05	+/-	0.00E+00
	PA1924	PA1924	1.60E+07	+/-	3.25E+06	1.00E+05	+/-	0.00E+00	1.00E+05	+/-	0.00E+00
	PA1925	PA1925	5.61E+07	+/-	1.31E+06	2.33E+07	+/-	3.21E+05	1.00E+05	+/-	0.00E+00
	PA2911	PA2911	3.57E+08	+/-	7.64E+06	2.35E+08	+/-	5.20E+06	5.79E+07	+/-	6.18E+06
	PA2912	PA2912	1.15E+08	+/-	3.21E+06	6.18E+07	+/-	1.39E+07	1.00E+05	+/-	0.00E+00
	PA2913	PA2913	2.06E+08	+/-	2.89E+06	1.35E+08	+/-	3.51E+06	8.34E+06	+/-	0.00E+00
	PA2914	PA2914									
	PA4063	PA4063	2.04E+09	+/-	6.56E+07	1.78E+09	+/-	4.93E+07	3.19E+08	+/-	1.85E+07
	PA4064	PA4064	2.68E+08	+/-	1.01E+07	1.69E+08	+/-	1.15E+07	2.26E+07	+/-	7.32E+06
	PA4065	PA4065	2.40E+08	+/-	2.23E+07	1.66E+08	+/-	7.94E+06	3.00E+07	+/-	5.20E+06
	PA4066	PA4066	1.30E+08	+/-	4.62E+06	9.66E+07	+/-	1.91E+06	1.00E+05	+/-	0.00E+00
	ZnuA	PA5498	2.68E+08	+/-	2.01E+07	2.09E+08	+/-	8.19E+06	2.34E+07	+/-	1.12E+07
	ZnuB	PA5501									
	ZnuC	PA5500	6.94E+07	+/-	2.14E+07	7.68E+07	+/-	0.00E+00	1.00E+05	+/-	0.00E+00
	ZnuD	PA0781	7.07E+09	+/-	3.67E+08	4.83E+09	+/-	2.86E+08	1.75E+09	+/-	1.39E+08
	ZrmA	PA4837	8.61E+08	+/-	9.61E+06	5.35E+08	+/-	3.26E+07	1.06E+08	+/-	5.13E+06
	ZrmB	PA4836	1.54E+08	+/-	5.03E+06	3.81E+07	+/-	5.83E+06	1.00E+05	+/-	0.00E+00
	ZrmC	PA4835	9.13E+07	+/-	3.33E+06	6.00E+07	+/-	5.45E+06	1.00E+05	+/-	0.00E+00
	ZrmD	PA4834	2.42E+07	+/-	0.00E+00	3.28E+07	+/-	0.00E+00	1.00E+05	+/-	0.00E+00
C+/C- paralogs	DksA	PA4723	1.25E+09	+/-	4.00E+07	9.26E+08	+/-	6.17E+07	1.12E+09	+/-	7.21E+07
	DksA2	PA5536	6.85E+08	+/-	8.38E+07	3.36E+08	+/-	4.65E+07	1.52E+07	+/-	2.14E+06
	RpmE	PA5049	2.09E+09	+/-	1.57E+08	2.26E+09	+/-	7.51E+07	2.25E+09	+/-	1.04E+08
	RpmE2	PA3601	2.48E+09	+/-	4.58E+07	6.77E+08	+/-	7.13E+08	1.00E+00	+/-	0.00E+00
	RpmJ	PA4242									
	RpmJ2	PA3600									
	PyrC	PA3527	2.62E+08	+/-	23180452	2.73E+08	+/-	8.62E+06	2.21E+08	+/-	3.37E+07
	PyrC2	PA5541	1.57E+08	+/-	8.89E+06	1.30E+08	+/-	8.39E+06	2.21E+07	+/-	7.01E+06
	FolE	PA3438	1.72E+08			2.09E+08			2.04E+08		
	FolE2	PA5539	6.03E+08	+/-	1.27E+07	3.84E+08	+/-	7.09E+06	1.39E+07	+/-	1.79E+06
Others	PA2807	PA2807	1.00E+05	+/-	0.00E+00	1.00E+05	+/-	0.00E+00	1.46E+08	+/-	2.76E+07
	OprD	PA0958	1.34E+09	+/-	1.53E+07	9.50E+08	+/-	1.64E+07	2.23E+08	+/-	1.55E+07
	PA5534	PA5534	6.17E+08	+/-	1.85E+07	3.85E+08	+/-	1.31E+07	1.00E+05	+/-	0.00E+00
	PA5535	PA5535	1.52E+09	+/-	6.24E+07	6.83E+08	+/-	8.54E+07	4.78E+07	+/-	4.40E+06
	PA5540	PA5540	4.25E+08	+/-	8.41E+07	2.75E+08	+/-	6.05E+07	1.60E+07	+/-	2.97E+06
