# Supplementary material for: Global Analysis of the Zinc Homeostasis Network in Pseudomonas aeruginosa and Its Gene Expression Dynamics
Source: Front Microbiol. 2021 Oct 8;12:739988. doi: 10.3389/fmicb.2021.739988 (PMC8531726; doi:10.3389/fmicb.2021.739988)
Supplement: Supplementary Figure 1 — PA2807 and metal resistance. Spot test assay of a serial dilution of the WT and the ΔPA2807 mutant carrying either an empty pMMB66EH plasmid (pE) or the PA2807 gene cloned under the inducible tac promoter of the pMMB66EH plasmid (pPA2807). After induction with 0.1 mM IPTG 10 μL of the various dilutions were spotted onto LB, LB + 3 mM CuCl2 or 5 mM ZnCl2, as indicated and incubated for 24 h at 37°C. [file Data_Sheet_1.zip › Data Sheet 1/Table 4.PDF]

Table S4: Strains and plasmids used in this study.

Strain or plasmid	Relevant characteristic(s)[a]	Reference or source
P. aeruginosa		
Wild type	PAO1 wild type	laboratory collection
ΔczcRΔczcS	PAO1 Δ*czcR*Δ*czcS*	(1)
ΔcopRΔcopS	PAO1 Δ*copR*Δ*copS*	This study
ΔPA2807	PAO1 Δ*PA2807*	This study
E. coli		
DH5α	recA1, endA1, hsdR17, deoR, thi-1, supE44, gyrA96, relA1, Δ(lacZYA-argF), U169(φ80dlacZΔM15)	(2)
BL21(DE3)	E. coli str. B F– ompT gal dcm lon hsdSB(rB–mB–) λ(DE3 [lacI lacUV5-T7p07 ind1 sam7 nin5]) [malB+]K-12(λS)	(3)
**Plasmids**		
pME3087	Suicide plasmid, Co1E1 replicon; Tc^r	(4)
pMMB66EH	Expressing vector carrying an IPTG-inducible promoter; Ap^r, Cb^r	(5)
pMMB66EH-*PA2807*	pMMB66EH derivative, carrying the *PA2807* gene; Ap^r, Cb^r	This study
pME6001	pME6000 derivative plasmid; Gm^r	(6)
pME6001-*PA2807*	pME6001 carrying the PA2807 gene and its promoter; Gm^r	This study
pBBR1-*gfp*	Transcriptional *gfp* fusion cloning vector; Ap^r, Cb^r	(7)
czcC:gfp	pBBR1 derivative, carrying the *czcC* promoter; Ap^r, Cb^r	(8)
czcD:gfp	pBBR1 derivative, carrying the *czcD* promoter; Ap^r, Cb^r	This study
pGex-2T-*zur*	GST-fusion expression plasmid, carrying the *zur* gene; Ap^r	This study

[a]Antibiotic resistance is indicated by r: Ap ampicillin; Tc tetracycline; Cb carbenicillin; Gm gentamycin

References:

1. Caille, O., Rossier, C. and Perron, K. (2007) A copper-activated two-component system interacts with zinc and imipenem resistance in Pseudomonas aeruginosa. *J Bacteriol*, **189**, 4561-4568.
2. Sambrook, J., and D. W. Russell. . (2001.) *Molecular cloning: a laboratory manual* 3rd ed.Cold Spring Harbor Laboratory Press, Cold Spring Harbor, NY.
3. Studier, F.W., Rosenberg, A.H., Dunn, J.J. and Dubendorff, J.W. (1990) Use of T7 RNA polymerase to direct expression of cloned genes. *Methods in enzymology*, **185**, 60-89.
4. Voisard, C., Bull, C.T., Keel, C., Laville, J., Maurhofer, M., Schnider, U., Défago, G. and Haas, D. (1994), *Molecular Ecology of Rhizosphere Microorganisms*, pp. 67-89.

5. Furste, J.P., Pansegrau, W., Frank, R., Blocker, H., Scholz, P., Bagdasarian, M. and Lanka, E. (1986) Molecular cloning of the plasmid RP4 primase region in a multi-host-range tacP expression vector. *Gene*, **48**, 119-131.
6. Blumer, C., Heeb, S., Pessi, G. and Haas, D. (1999) Global GacA-steered control of cyanide and exoprotease production in Pseudomonas fluorescens involves specific ribosome binding sites. *Proc Natl Acad Sci U S A*, **96**, 14073-14078.
7. Ouahrani-Bettache, S., Porte, F., Teyssier, J., Liautard, J.P. and Kohler, S. (1999) pBBR1-GFP: a broad-host-range vector for prokaryotic promoter studies. *Biotechniques*, **26**, 620-622.
8. Ducret, V., Gonzalez, M.R., Leoni, S., Valentini, M. and Perron, K. (2020) The CzcCBA Efflux System Requires the CadA P-Type ATPase for Timely Expression Upon Zinc Excess in Pseudomonas aeruginosa. *Front Microbiol*, **11**, 911.
