# Supplementary material for: Global Analysis of the Zinc Homeostasis Network in Pseudomonas aeruginosa and Its Gene Expression Dynamics
Source: Front Microbiol. 2021 Oct 8;12:739988. doi: 10.3389/fmicb.2021.739988 (PMC8531726; doi:10.3389/fmicb.2021.739988)
Supplement: Supplementary Figure 1 — PA2807 and metal resistance. Spot test assay of a serial dilution of the WT and the ΔPA2807 mutant carrying either an empty pMMB66EH plasmid (pE) or the PA2807 gene cloned under the inducible tac promoter of the pMMB66EH plasmid (pPA2807). After induction with 0.1 mM IPTG 10 μL of the various dilutions were spotted onto LB, LB + 3 mM CuCl2 or 5 mM ZnCl2, as indicated and incubated for 24 h at 37°C. [file Data_Sheet_1.zip › Data Sheet 1/Table 5.PDF]

Table S5: Primers used in this study.

Amplicon	PA number	Primer	Sequence 5'-3'	Position (from the AUG)	Length
**Coding regions**					
czcC	PA2522	czcC1	GGTCAGCATCGGCAGCAAGTACG	834	206
		czcC2	GGTCGTAGGCCTGTACCGCTTCG	1039	
czcD	PA0397	574	GGCGTGGCCTTCTATATCCT	285	183
		575	TCCAGACCTCCAGGTAGGC	450	
ptrA / c	PA2808	754	CGTACCTTCACTGCCTCCAC	502	176
		755	TTGTCCTTCTTCGTCGCTTC	179	
	PA2807	576	GGTGCTCGGCGATATGTACT	212	158
		577	GCATCTCCAGCATCTCCTTC	350	
oprD	PA0958	oprD1	ATCTACCGCACAAACGATGAAGG	772	156
		oprD2	GCCGAAGCCGATATAATCAAACG	927	
oprF	PA1777	594	GGTTACTTCCTGACCGACGA	172	209
		595	TCGCTGTTGATGTTGGTGAT	380	
a	PA2807	1338	TTGACCTCGATCGCCCGAGG	238	222
		1339	ACAGGATAGGCGACATCAAC	35	
b	PA2807-08	1340	AGGCGGCGCGGAAACATACG	70	318
		1341	ACCAGGAGAAACTGGCGAAG	134	
**Promoter regions**					
pznuD	PA0781	1254	ACTTTCCCGTTCCTCTTCGC	-215	197
		1255	TGGGTCGGACTTTTTTCAGG	-18	
pPA1922	PA1922	1256	GAGCGCCATGATCTGCTCGG	-197	272
		1257	GAAGCAGGTAGAGGAACGGC	75	
pPA2439	PA2439	1344	GTGCTGCACACAGACTCTCG	-140	167
		1345	GTCCAGGTCGACTCTCATCT	17	
pPA2911	PA2911	1258	CATAGCAGGCCGGCGTGAAG	-458	229
		1259	TCACCGAAGTGACGCACCGG	-229	
pPA4063	PA4063	1027	GTTTCTCCACAAGCGAATCG	-4	182
		1028	ATGGGCCTGGGCGGCGAACG	-186	
pzrmA	PA4837	1109	GGCTGGGCTGGTCGTCGGA	-141	225
		1110	CCGCGGGACTTTCCGTTTCC	84	
pznuA	PA5498	969	CTCTGCCAGACCAGTTCCAG	-227	214
		970	TGAAAGGTGGTTAGCGGGTA	-14	
prpmE2	PA3600	1107	TCGAGCAGCCGAATGGTCGC	-99	182
		1108	GGTGGAGCCGATCAGGAAGT	83	
pdksA2	PA5536	1105	AGTAAAGGCGGAGGACGCGG	-137	203
		1106	AGAAGTCCTGCTGGGCTTCG	66	
**DNA cloning**					
**Feature**		**Primer**	**Sequence 5'-3'**	**Length**	

ΔPA2807	588	GCCgaattcCATGTTCATCCCTCGTTACA	489	
	589b	GCCggtaccACATCTTTGTCAGCTTGCCG		
	590b	GCCggtaccCGATCCGTATACACTTGCCG	425	
	591	GCCggatccGGTCTTTCTGCAATACCGCG		
ΔcopRS	715	CCCgaattcCTGTCGGGAGCATTACATCT	491	
	716	CCCggtaccTCGTTACATTTGCCGGCCAT		
	717	CCCggtaccAACGCGAAGCCCCCATGACG	393	
	718	CCCggatccGTGCTGGTGGTGGTGCTTAC		
pMMB66EH-PA2807	626	GCCgaattcATGCTCCCGACAGCCAGCCG	639	
	627	GCCggatccTTCACGCCGGCCGTCCGCCG		
pME6001- PA2807::6His	1309	CGCggatccTCAGCAGTTTCATGTTCATC	1115	
	1310	CGCaagcttTCA**atgatgatgatgatgatg**GGGCTGCACGGTCAGTTGAC		
pGEX2T-zur	1044	GCGggatccATGTACAAGATTGCGCCCAAGACCC	503	
	1045	GCCgaattcTCAGGCGTCCTTCTGGTCCC		

Restriction sites are underlined (lowercase) and 6His tag is indicated as lowercase in bold
