# Supplementary figures and images for: Global Analysis of the Zinc Homeostasis Network in Pseudomonas aeruginosa and Its Gene Expression Dynamics
Source: Front Microbiol. 2021 Oct 8;12:739988. doi: 10.3389/fmicb.2021.739988 (PMC8531726; doi:10.3389/fmicb.2021.739988)

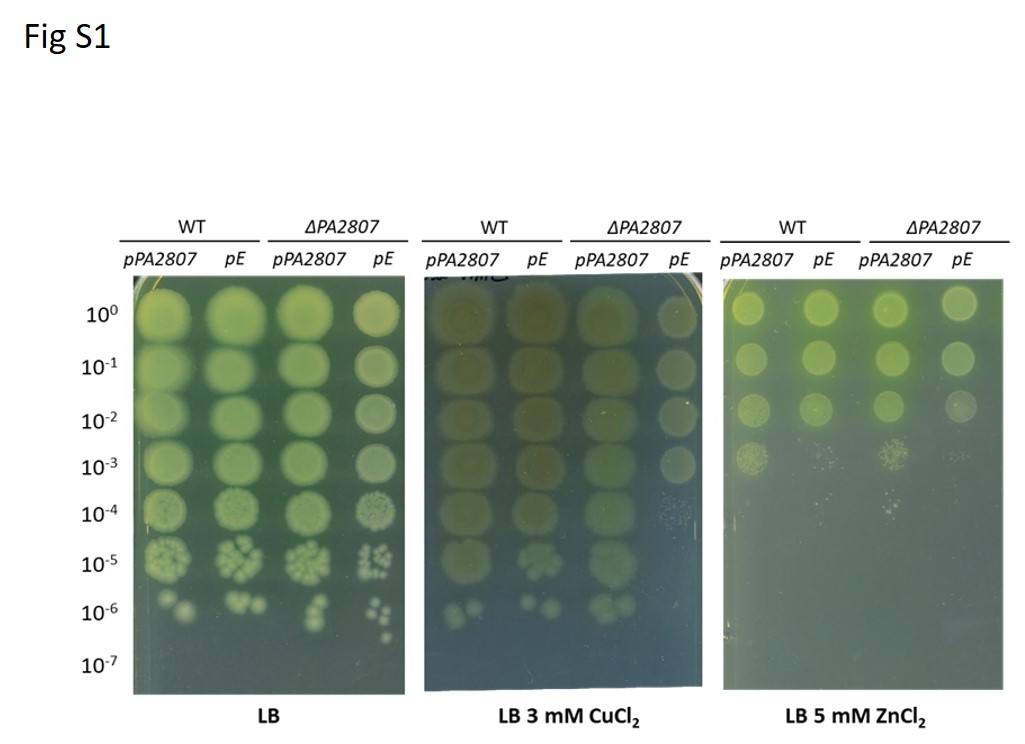

Supplement: Supplementary Figure 1 — PA2807 and metal resistance. Spot test assay of a serial dilution of the WT and the ΔPA2807 mutant carrying either an empty pMMB66EH plasmid (pE) or the PA2807 gene cloned under the inducible tac promoter of the pMMB66EH plasmid (pPA2807). After induction with 0.1 mM IPTG 10 μL of the various dilutions were spotted onto LB, LB + 3 mM CuCl2 or 5 mM ZnCl2, as indicated and incubated for 24 h at 37°C. [file Data_Sheet_1.zip › Data Sheet 1/Figure 1.JPEG]

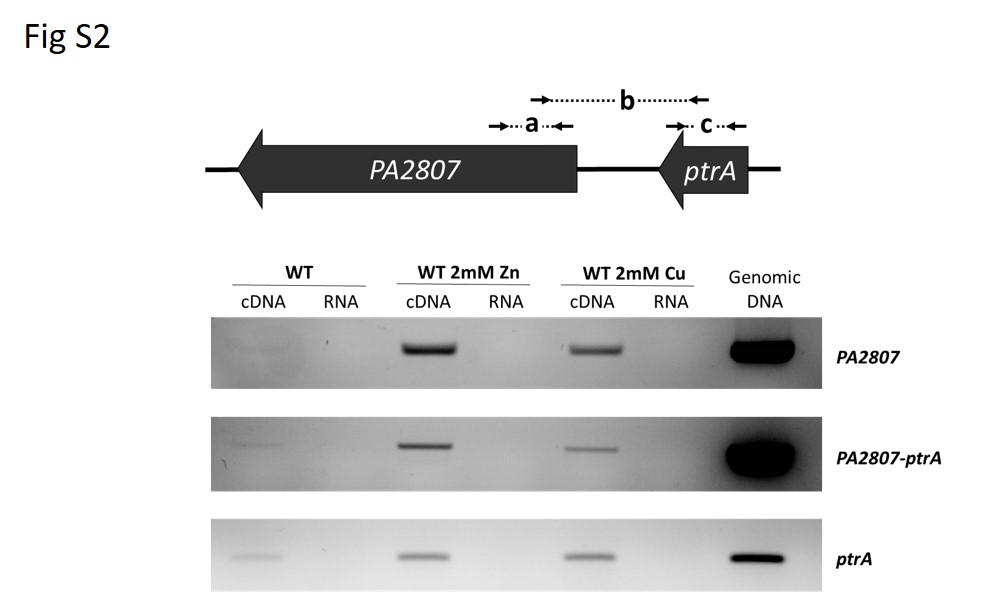

Supplement: Supplementary Figure 1 — PA2807 and metal resistance. Spot test assay of a serial dilution of the WT and the ΔPA2807 mutant carrying either an empty pMMB66EH plasmid (pE) or the PA2807 gene cloned under the inducible tac promoter of the pMMB66EH plasmid (pPA2807). After induction with 0.1 mM IPTG 10 μL of the various dilutions were spotted onto LB, LB + 3 mM CuCl2 or 5 mM ZnCl2, as indicated and incubated for 24 h at 37°C. [file Data_Sheet_1.zip › Data Sheet 1/Figure 2.JPEG]

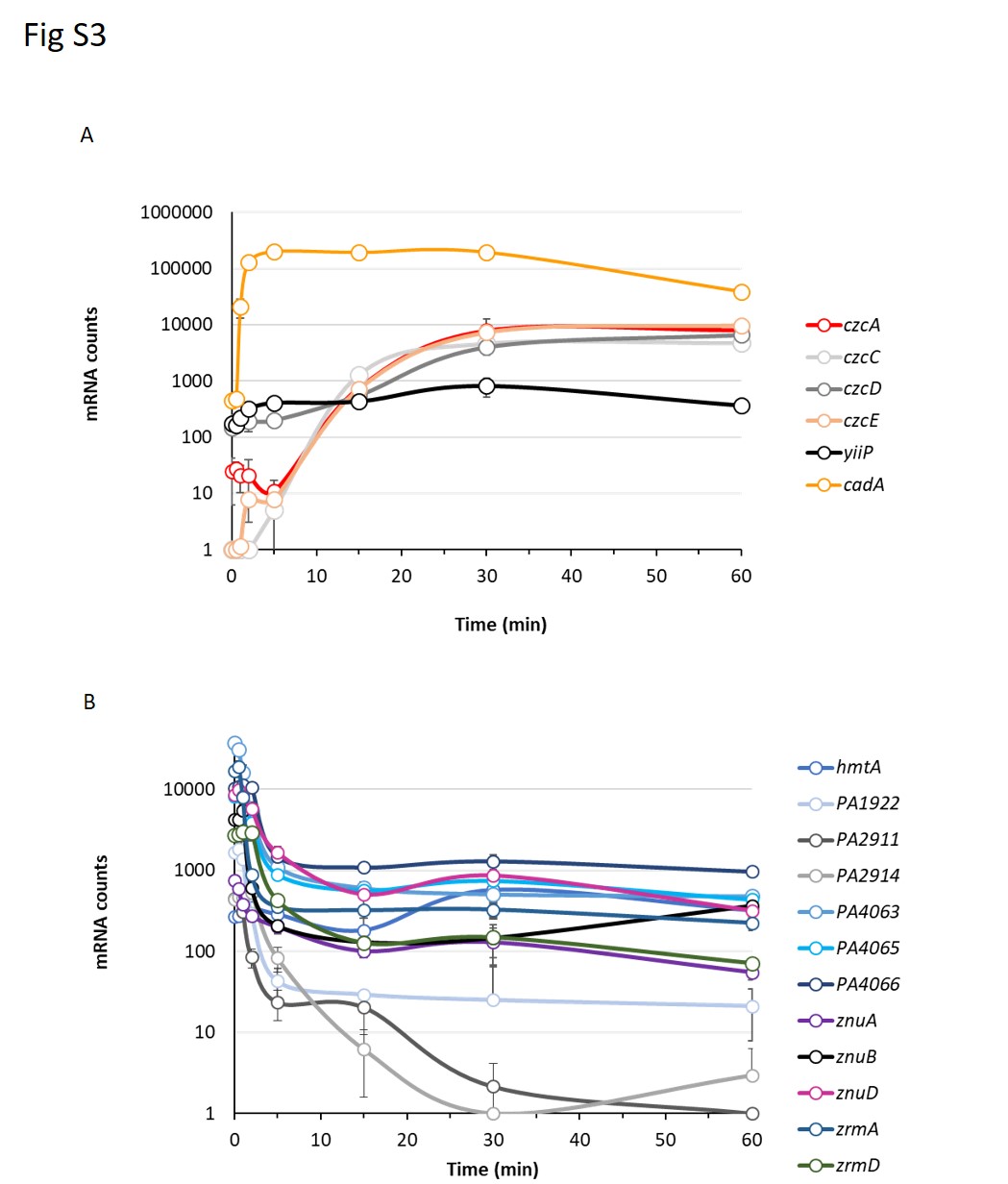

Supplement: Supplementary Figure 1 — PA2807 and metal resistance. Spot test assay of a serial dilution of the WT and the ΔPA2807 mutant carrying either an empty pMMB66EH plasmid (pE) or the PA2807 gene cloned under the inducible tac promoter of the pMMB66EH plasmid (pPA2807). After induction with 0.1 mM IPTG 10 μL of the various dilutions were spotted onto LB, LB + 3 mM CuCl2 or 5 mM ZnCl2, as indicated and incubated for 24 h at 37°C. [file Data_Sheet_1.zip › Data Sheet 1/Figure 3.JPEG]

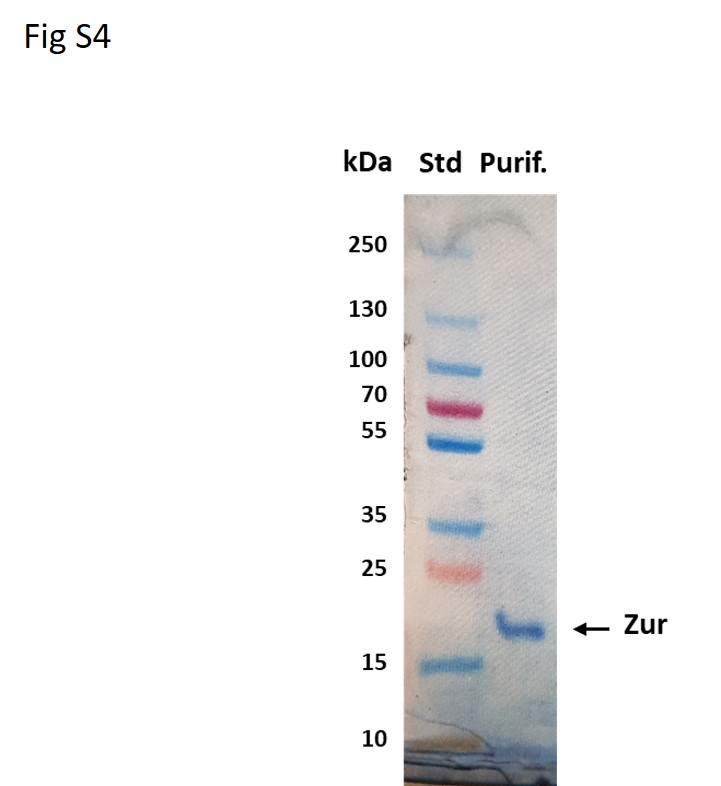

Supplement: Supplementary Figure 1 — PA2807 and metal resistance. Spot test assay of a serial dilution of the WT and the ΔPA2807 mutant carrying either an empty pMMB66EH plasmid (pE) or the PA2807 gene cloned under the inducible tac promoter of the pMMB66EH plasmid (pPA2807). After induction with 0.1 mM IPTG 10 μL of the various dilutions were spotted onto LB, LB + 3 mM CuCl2 or 5 mM ZnCl2, as indicated and incubated for 24 h at 37°C. [file Data_Sheet_1.zip › Data Sheet 1/Figure 4.JPEG]
